# Exosomal circ-ZNF652 Mediates TGF-β1-Induced Epithelial-Mesenchymal Transition and Glycolytic Reprogramming to Promote Hepatocellular Carcinoma Progression

**DOI:** 10.4014/jmb.2606.06001

**Published:** 2026-09-02

**Authors:** Xianli Niu, Xiaomin Zhang, Guangxia Liang, Nina Wei, Haibo Peng, Xiangyang Li

**Affiliations:** 1 Guangdong Jiangmen College of Chinese Medicine, Jiangmen, Guangdong 529000, P. R. China; 2 Guangdong Provincial People’s Hospital Zhuhai Hospital (Jinwan Central Hospital of Zhuhai), Zhuhai, Guangdong 519000, P. R. China; 3 The Fifth Affiliated (Zhuhai) Hospital of Zunyi Medical University, Zhuhai, Guangdong 519100, P. R. China; 4 Zhuhai Campus of Zunyi Medical University, Zhuhai, Guangdong 519041, P. R. China

**Keywords:** Hepatocellular carcinoma, Exosomal circ-ZNF652, Transforming growth factor-β1 (TGF-β1), Epithelial-mesenchymal transition (EMT), Glycolytic reprogramming, Cell proliferation, AKT/HIF-1α signaling pathway

## Abstract

Hepatocellular carcinoma (HCC) progression involves epithelial-mesenchymal transition (EMT) and glycolytic reprogramming induced by TGF-β1. The role of exosomal circular RNAs in coordinating these processes remains unclear. This study aims to investigate whether exosomal circ-ZNF652 is associated with TGF-β1-induced mesenchymal marker induction and glycolysis in HCC and elucidate the underlying mechanism. Exosomes were isolated from SMMC-7721 cells and characterized by TEM, NTA, and CD63 western blot. RT-qPCR measured circ-ZNF652 expression. Cell viability, migration, cycle, and apoptosis were assessed by MTT, wound healing, and flow cytometry. EMT and glycolysis proteins were detected by western blot. Glucose uptake, lactate, and HK/LDHA activities were measured. *In vivo* xenograft and rescue experiments with AKT/HIF-1α inhibitors were performed. TGF-β1 stimulation upregulated circ-ZNF652 in SMMC-7721 cells and their exosomes. Exosomal circ-ZNF652 was internalized by HCC cells and further enhanced TGF-β1-induced upregulation of EMT markers (Twist1, N-cadherin, vimentin, MMP-9). It also promoted cell proliferation, migration, S-phase entry, reduced apoptosis and Caspase-3 activity. Moreover, Exos circ-ZNF652 augmented TGF-β1-induced glycolysis as evidenced by increased glucose uptake, lactate secretion, HK/LDHA activities, and Glut-1/HIF-1α expression. *In vivo*, circ-ZNF652 overexpression significantly increased tumor volume (1286.5 vs 452.8 mm^3^) and weight (1.32 vs 0.48 g), along with elevated Ki-67, N-cadherin, HIF-1α, and p-AKT. Rescue experiments showed that AKT or HIF-1α inhibition reversed circ-ZNF652-mediated EMT and glycolysis. All differences were statistically significant (*P*<0.05). To sum up, exosomal circ-ZNF652 is associated with TGF-β1-induced mesenchymal phenotype acquisition and glycolysis via the AKT/HIF-1α pathway, promoting HCC progression. It represents a potential non-invasive biomarker and therapeutic target for HCC.

## Introduction

Hepatocellular carcinoma (HCC), which accounts for approximately 90% of primary liver malignancies, remains the third leading cause of cancer-related death globally, with a 5-year overall survival rate of less than 20% [1]. The poor prognosis of HCC is mainly attributed to early distant metastasis, postoperative recurrence and the occurrence of metabolic reprogramming, which enables tumor cells to adapt to the hypoxic tumor microenvironment (TME) and support rapid proliferation [2]. Therefore, elucidating the key molecular mechanisms underlying HCC metastasis and metabolic reprogramming is critical for developing novel diagnostic markers and targeted therapeutic strategies [3-5].

Epithelial-mesenchymal transition (EMT) is a highly conserved cellular process that converts epithelial cells into mesenchymal-like cells, and is a core event driving tumor invasion and metastasis [6]. In HCC, EMT is characterized by the downregulation of epithelial markers (*e.g.*, E-cadherin) and upregulation of mesenchymal markers (*e.g.*, N-cadherin, vimentin), as well as the activation of EMT-related transcription factors such as Twist1 and Snail [7]. Transforming growth factor-β1 (TGF-β1), a multifunctional cytokine in the TME, is a classic and potent inducer of EMT in HCC, and can activate the Smad and non-Smad signaling pathways to regulate the expression of EMT-related genes [8, 9]. Notably, recent studies have demonstrated that TGF-β1 not only induces EMT but also modulates tumor metabolic reprogramming, especially the Warburg effect (aerobic glycolysis), a hallmark of tumor cells in which glycolysis is enhanced even under normoxic conditions [10, 11]. This cross-regulation of EMT and glycolysis by TGF-β1 suggests a potential common molecular regulator in HCC, but the specific mechanism remains largely unclear [12].

Exosomes, small extracellular vesicles with a diameter of 30-150 nm, are key mediators of intercellular communication in the TME by transporting bioactive molecules including circular RNAs (circRNAs), microRNAs (miRNAs) and proteins [13]. Exosome-derived non-coding RNAs are stably expressed in body fluids and tumor cells, and play crucial roles in regulating HCC cell proliferation, EMT and metabolic reprogramming [14]. CircRNAs are a class of non-coding RNAs with covalently closed circular structures, which exhibit high structural stability and tissue/cell specificity compared with linear RNAs [15]. Due to these unique characteristics, circRNAs have been identified as potential diagnostic and prognostic biomarkers for various cancers including HCC [16]. Accumulating evidence has shown that circRNAs can mediate TGF-β1-induced EMT in tumors; for example, circ_0000512 promotes TGF-β1-induced EMT in non-small cell lung cancer by targeting the miR-145-5p/FOSL2 axis [17]. However, whether exosome-derived circRNAs mediate the TGF-β1-driven cross-talk between EMT and glycolytic reprogramming in HCC, and the underlying mechanisms, remain largely unexplored [14].

Circ-ZNF652, encoded by the ZNF652 gene on chromosome 19, has been reported to act as an oncogenic circRNA in HCC [18]. Li *et al*. previously reported that intracellular circ-ZNF652 promotes HCC cell proliferation and metastasis via modulating the miR-29a-3p/GUCD1 axis, and that its upregulation is closely associated with unfavorable prognosis in HCC patients [18]. Preliminary RNA sequencing of TGF-β1-stimulated SMMC-7721 cells identified circ-ZNF652 as one of the most significantly upregulated circRNAs (fold change = 2.3, P<0.01), consistent with its reported role in enhancing EMT in gastrointestinal cancers [19]. TGF-β1 is a key driver of EMT and exosomes often encapsulate bioactive molecules from parental cells. Given that AKT signaling integrates TGF-β1–driven EMT and metabolic reprogramming in HCC, and that HIF-1α acts as a key AKT downstream transcriptional driver of glycolysis, we hypothesized that the AKT/HIF-1α axis might underlie circ-ZNF652–mediated phenotypes [9, 20]. However, whether circ-ZNF652 is secreted into the extracellular space via exosomes, and whether Exos circ-ZNF652 is associated with TGF-β1-induced EMT and glycolysis in HCC, has not been reported to date. Hence, we hypothesized that circ-ZNF652 might be selectively packaged into exosomes upon TGF-β1 exposure, potentially mediating intercellular communication during EMT.

In this study, we isolated and identified exosomes from human HCC SMMC-7721 cells, and systematically investigated the expression of circ-ZNF652 in cells and exosomes after TGF-β1 stimulation. We further explored the effects of Exos circ-ZNF652 on TGF-β1-induced EMT, cell proliferation, migration and glycolysis in SMMC-7721 cells using a series of *in vitro* cell experiments, and verified the oncogenic role of circ-ZNF652 *in vivo* via nude mouse xenograft model. We also clarified the underlying molecular mechanism of circ-ZNF652 through rescue experiments. Our findings reveal a novel mechanistic link between TGF-β1 signaling, exosomal circRNAs, and the coordinated regulation of EMT and metabolic reprogramming in HCC, and highlight exosomal circ-ZNF652 as a promising molecular target for HCC diagnosis and targeted therapy.

## Materials and Methods

### Experimental Materials

Human HCC SMMC-7721 cells were purchased from the Shanghai Cell Bank of the Chinese Academy of Sciences. Exosome extraction kit, Exo-Red exosome fluorescent labeling reagent (Shanghai Future Industrial Co., Ltd., China); DMEM medium, RPMI-1640 medium, fetal bovine serum (FBS) (Shanghai Jingkang Bioengineering Co., Ltd., China); TEM detection service (Annuo Lun (Beijing) Biotechnology Co., Ltd., China); Antibodies against CD63, Twist1, N-cadherin, vimentin, MMP-9, Glut-1, HIF-1α, Ki-67, p-AKT (Abcam, UK); HK detection kit, LDHA detection kit (Shanghai Bingqing Biotechnology Co., Ltd., China); PI Staining Kit, Annexin V-FITC/PI Apoptosis Detection Kit (Beyotime, China); Glucose Uptake Assay Kit (ab136955, Abcam, UK); Lactate Assay Kit (ab65331, Abcam, UK); S-P immunohistochemistry kit (Beijing Zhongshan Biotechnology Co., Ltd., China); MTT reagent, DMSO (Sigma, USA); RNA extraction kit (QIAGEN, Germany); RT-qPCR kit, PrimeScript RT Reagent Kit with gDNA Eraser (TaKaRa, Japan); AKT inhibitor (MK-2206), HIF-1α inhibitor (KC7F2) (Selleck, USA); ECL Plus Western Blotting Substrate (Thermo Fisher Scientific, USA). Multiskan FC microplate reader (Beijing Yiaoke Biotech Co., Ltd., China); Flow cytometer (Shanghai Yuanye Biotechnology Co., Ltd., China); Fluorescence microscope, inverted microscope (Olympus IX73, Japan); Transmission electron microscope (JEM-1400Plus, JEOL, Japan); Nanoparticle tracking analysis (NTA) instrument (ZetaView PMX 110, Particle Metrix, Germany); Western blot electrophoresis and transfer system, ChemiDoc XRS+ System (Bio-Rad, USA).

## Experimental Methods

### Cell Culture and Grouping

Preparation of exosome-depleted FBS: FBS was ultracentrifuged at 100,000 × g overnight at 4°C. The supernatant was retained as exosome-depleted FBS.

Cell culture conditions: Hypoxia group: SMMC-7721 cells were seeded in RPMI-1640 medium supplemented with 10% exosome-depleted FBS and cultured in a hypoxic incubator (1% O_2_, 5% CO_2_) at 37°C for 48 h. Normoxia group: Cells were seeded in DMEM medium supplemented with 10% exosome-depleted FBS and cultured in a standard incubator (5% CO_2_) at 37°C. TGF-β1 (Peprotech, USA) was dissolved in sterile PBS containing 0.1% BSA to a stock concentration of 1 g/L, and diluted to the working concentration of 0.005 g/L before use.

Experimental groups: Based on TGF-β1 stimulation and circ-ZNF652 intervention, cells were divided into 4 groups with three biological replicates for each group: (1)Blank control group (Con): No TGF-β1 stimulation, no circ-ZNF652 intervention; (2) TGF-β1 group: Stimulated with 0.005 g/L TGF-β1 for 48 h; (3) TGF-β1 + NC RNA group: TGF-β1 stimulation + transfection with negative control RNA for 48 h; (4)TGF-β1 + Exos-circ-ZNF652 group: TGF-β1 stimulation + Exos circ-ZNF652 intervention for 48 h. Exosome doses were normalized to total protein concentration quantified by BCA assay (Thermo Fisher), and cells were treated with exosomes at 50 μg/mL (final concentration) in serum-free medium for 24 h, unless otherwise indicated.

BALB/c nude mice (4-6 weeks old, female) were randomly divided into 3 groups (6 mice per group): (1) Control group: Subcutaneous injection of empty vector SMMC-7721 cells (1×10^6^ cells/mouse); (2) OE-circ group: Subcutaneous injection of circ-ZNF652 overexpression SMMC-7721 cells (1 × 10^6^ cells/mouse); (3) KD-circ group: Subcutaneous injection of circ-ZNF652 knockdown SMMC-7721 cells (1 × 10^6^ cells/mouse)

**Exosome extraction and fluorescent labeling.** (1) Exosome extraction: SMMC-7721 cells (1 × 10^5^/well) were seeded in 6-well plates. After overnight attachment, the medium was replaced with serum-free medium containing exosome-depleted FBS, and cells were cultured for another 48 h. The supernatant from three replicate wells (total 5 mL) was collected. The collected supernatant was first centrifuged at 300 × g for 10 min, 2000 × g for 20 min, and 10,000 × g for 30 min at 4°C to remove cell debris and microvesicles; then the supernatant was mixed with the exosome extraction kit reagent (1:1, v/v) and incubated overnight at 4°C, followed by centrifugation at 10,000 × g for 10 min at 4°C to obtain exosome pellets, which were resuspended in PBS (designated as PBS-Exos).

Fluorescent labeling: Exosomes were mixed with Exo-Red reagent at a volume ratio of 10:1 and incubated at 37°C for 10 min. A 100 μL termination solution was added to stop the reaction, followed by incubation at 4°C for 30 min. The mixture was centrifuged at 14000 × g for 3 min, and the labeled exosomes were resuspended in 200 μL PBS. These were then co-incubated with SMMC-7721 cells in 24-well plates for 48 h (Exos-Trans group), and fluorescence intensity was observed under a fluorescence microscope (×100 magnification).

**Exosome identification.** (1) TEM observation: A 30 μL drop of exosome suspension was placed on a copper grid, allowed to settle for 1 min, and excess liquid was removed with filter paper. A 30 μL drop of phosphotungstic acid solution (pH 6.8) was added for negative staining at room temperature for 5 min. After drying, the grid was observed and imaged under TEM (scale bar = 100 nm).

(2) Nanoparticle tracking analysis (NTA): NTA was performed using a ZetaView PMX 110 to determine the particle size distribution and concentration of exosomes. The exosome suspension was diluted 100-fold with sterile PBS, and three replicates were detected for each sample; the data were analyzed using ZetaView 8.02.28 software.

Western blot detection of CD63: A 200 μL aliquot of concentrated exosomes was centrifuged at 14000 ×g for 3 min. Exosomes were lysed with RIPA lysis buffer on ice for 15 min, centrifuged at 14000 × g for 15 min, and the supernatant (total protein) was collected. Protein concentration was quantified using the BCA method. SDS-PAGE was performed (30 μg protein/lane), followed by transfer to a PVDF membrane. The membrane was blocked with 5% skim milk for 1 h, incubated with primary antibody against CD63 (1:1000 dilution) overnight at 4°C, washed three times with TBST, incubated with HRP-conjugated secondary antibody (1:2000 dilution) at room temperature for 1 h, and visualized using ECL chemiluminescence. Gray value analysis was performed using ImageJ software. Exosome characterization was performed according to MISEV2018 guidelines. Protein markers were analyzed by Western blotting using antibodies against CD9, CD81, TSG101, Alix (positive markers), and Calnexin (negative marker for intracellular/ER contamination).

**RT-qPCR detection of circ-ZNF652 expression.** RNA extraction: Total RNA from cells and exosomes of each group was extracted using the QIAGEN RNeasy Mini Kit according to the manufacturer's instructions.

RT-qPCR reaction: Total RNA (1 μg) was reverse-transcribed into cDNA using a PrimeScript RT Reagent Kit with gDNA Eraser according to the manufacturer's instructions; the reaction conditions were 42°C for 2 min (gDNA erasure), 37°C for 15 min (reverse transcription), and 85°C for 5 s (enzyme inactivation). Using GAPDH as an internal reference, amplification was performed on a Bio-Rad RT-qPCR instrument. Reaction conditions: 95°C pre-denaturation for 30 s; 35 cycles of 95°C denaturation for 5 s, 60°C annealing/extension for 20 s. All primers were synthesized by Sangon Biotech (China), and the primer sequences were as follows: circ-ZNF652 forward: 5'-GGGCACAAACAGTTCATGTG-3', reverse: 5'-TGCGTTTGAATGATTTTCCA-3'; GAPDH forward: 5'-ACCCAGAAGACTGTGGATGG-3', reverse: 5'-TTCAGCTCAGGGATGACCTT-3'. Result calculation: The relative expression level of circ-ZNF652 was calculated using the 2^(^−ΔΔCt^) method.

**Cell Viability Assay (MTT).** Cells from each group were seeded in 96-well plates at 100 μL/well (density 1×10^4^ cells/well), with 9 replicate wells per group (3 technical replicates × 3 biological replicates). After 24 h incubation at 37°C in a 5% CO_2_ incubator, all groups were cultured with fresh complete medium for another 48 h. Then 20 μL of MTT solution (5 mg/mL) was added to each well. After 4 h, the supernatant was discarded, and 150 μL DMSO was added to dissolve the formazan crystals. Absorbance (A value) was measured at 570 nm using a microplate reader. Cell growth inhibition rate (IR) was calculated: IR (%) = (1 - A value of experimental group / A value of control group) × 100%.

**Flow cytometry analysis of cell cycle and apoptosis.** Logarithmic growth phase cells (1 × 10^5^/mL) from each group were collected, seeded in 100 mL culture flasks, and cultured at 37°C for 24 h. Then, the corresponding intervention was performed for 48 h. Cells were digested with 0.1% trypsin and washed twice with PBS. At least 10,000 cells were collected for each sample.

Cell cycle analysis: Cells were fixed in 70% ice-cold ethanol at 4°C for 1 h. After PBS washing, an equal volume of PI staining solution (PI + RNase) was added, and cells were incubated at 4°C in the dark for 30 min. Cell cycle distribution (G_0_/G_1_, S, G_2_/M phases) was analyzed using a flow cytometer (excitation wavelength 488 nm) and ModFit software.

Apoptosis analysis: Cells were stained with Annexin V-FITC/PI apoptosis detection kit according to the manufacturer's instructions, and apoptosis rate was analyzed using a flow cytometer.

**Wound healing assay for migration ability.** Cells from each group were seeded in 6-well plates at 2 × 10^5^/well. After 12 h of culture, a straight wound line was created in the center of each well using a 200 μL pipette tip (ensuring uniform scratch width). The initial wound width (0h) was photographed under an inverted microscope (× 100 magnification). The medium was then replaced with serum-free medium, and cells were cultured for another 24 h. The wound width was photographed again. The relative migration value was calculated: Relative migration value = (0 h width experimental group - 24 h width experimental group) / (0 h width control group - 24 h width control group). The experiment was repeated three times, each with three replicate wells.

**Western blot analysis of EMT and glucose metabolism-related proteins.** Total protein was extracted from cells and tumor tissues of each group (as described in 2.2.3) to detect the expression of EMT-related proteins (Twist1, N-cadherin, vimentin, MMP-9) and glucose metabolism-related proteins (Glut-1, HIF-1α), as well as Ki-67, and p-AKT. Primary antibody dilutions: Twist1 (1:1000), N-cadherin (1:1000), vimentin (1:2000), MMP-9 (1:1000), Glut-1 (1:1000), HIF-1α (1:1000), Ki-67 (1:1000), p-AKT (1:1000), GAPDH (1:5000). Subsequent steps were the same as in 2.2.3. Gray values were analyzed using ImageJ software (relative expression was calculated using GAPDH as an internal reference).

**Detection of glycolytic indices.** Glucose uptake and lactate secretion: Glucose uptake and lactate production were measured using the Glucose Uptake Assay Kit and Lactate Assay Kit according to the manufacturer's instructions. Glucose uptake was measured by detecting the fluorescence intensity at Ex/Em = 535/587 nm and calculated as the difference between the initial and remaining glucose concentrations in the culture medium; lactate secretion was measured by absorbance at 570 nm.

HK/LDHA activity: Cells were seeded in 24-well plates at 2 × 10^5^/well. After culture and intervention, the medium was discarded, and cells were washed twice with PBS. A 150 μL aliquot of HK/LDHA release reagent (diluted 10-fold) was added, and cells were incubated at 37°C for 1 h. Following the kit instructions, OD values were measured at 563 nm (HK) and 490 nm (LDHA) using a microplate reader. Enzyme activity was calculated based on standard curves. Each sample was measured in triplicate.

Tumor tissue glycolytic indices: Tumor tissues were homogenized with precooled PBS, and the supernatant was collected after centrifugation. Lactate content and HK activity in tumor tissues were detected using the corresponding kits according to the manufacturer's instructions.

**BrdU ELISA and caspase-3 activity assay.** BrdU ELISA assay: Cell proliferation activity (DNA synthesis) was detected using BrdU ELISA Kit according to the manufacturer's instructions, and the absorbance was measured at 450 nm using a microplate reader.

Caspase-3 activity assay: Caspase-3 activity in cells was detected using Caspase-3 Colorimetric Assay Kit according to the manufacturer's instructions. The absorbance was measured at 405 nm, and the relative activity was calculated with the blank control group as 100%.

**Rescue experiment.** SMMC-7721 cells were transfected with circ-ZNF652 overexpression vector to construct circ-ZNF652 overexpression cell line (OE-circ). Then OE-circ cells were divided into 3 groups: (1) OE-circ group: No inhibitor treatment; (2) OE-circ + MK-2206 group: Treated with 10 μmol/L AKT inhibitor MK-2206; (3) OE-circ + KC7F2 group: Treated with 20 μmol/L HIF-1α inhibitor KC7F2. After 48 h of treatment, total protein was extracted, and the expression of EMT-related (Twist1, N-cadherin, vimentin) and glycolysis-related (Glut-1, HIF-1α) proteins was detected by Western blot (as described in 2.2.8).

**Animal xenograft experiment.** BALB/c nude mice were raised under specific pathogen-free (SPF) conditions. Corresponding SMMC-7721 cells (1×10^6^ cells/mouse) were subcutaneously injected into the right axilla of nude mice (6 mice per group). Tumor volume was measured weekly using a vernier caliper, and tumor volume was calculated as: Volume (mm^3^) = length × width^2^ / 2. After 4 weeks, the mice were sacrificed, and the tumors were dissected and weighed. Tumor tissues were stored at -80°C for subsequent Western blot and glycolytic index detection.

### Statistical Analysis

All statistical analyses were performed using SPSS 19.0 (IBM Corp., USA) and GraphPad Prism 9.0 (GraphPad Software, USA). Normally distributed continuous data are presented as mean ± standard deviation (Mean ± SD) unless specified otherwise. Comparisons among groups were performed using one-way analysis of variance (ANOVA) followed by Tukey’s post hoc test for multiple comparisons. Biological replicates refer to independent experiments using distinct cell passages or animal cohorts (n ≥ 3 for all *in vitro*/*vivo* assays). Technical replicates denote parallel measurements within the same sample (*e.g.*, triplicate wells for MTT assay). A two-sided *P*-value < 0.05 was considered statistically significant. Significance levels are denoted as follows: * *P* < 0.05, ** *P* < 0.01, *** *P* < 0.001 vs. the blank control (Con) group; # *P* < 0.05, ## *P* < 0.01, ### *P* < 0.001 vs. the TGF-β1 alone group.

## Results

### Identification and Cellular Uptake of Exosomes Derived from SMMC-7721 Cells

TEM revealed that the extracted vesicles exhibited typical cup-shaped or circular structures with diameters of approximately 30−150 nm, consistent with the morphological characteristics of exosomes (Fig. 1A). Western blot analysis showed positive expression of the exosome-specific marker CD63 in the exosome fraction, while its expression was very weak in the control group (cell lysate), confirming successful exosome isolation with high purity (Fig. 1B). Western blotting per MISEV2018 confirmed exosomal identity, showing enrichment of CD9, CD81, TSG101, and Alix, alongside the absence of organelle markers Calnexin and GM130 (Fig. 1C). These data verify high vesicle purity and exclude intracellular contamination. NTA analysis showed that the particle size of the extracted vesicles was mainly distributed in the range of 30−150 nm, which further confirmed the size characteristics of exosomes (Fig. 1D). Under fluorescence microscopy, red fluorescence (from Exo-Red labeled exosomes) was observed inside SMMC-7721 cells in the Exos-Trans group, indicating effective uptake of the extracted exosomes by the cells. These results collectively fulfill the MISEV-recommended criteria for exosome identification, supporting the validity of our isolated exosomes for subsequent functional assays. RT-qPCR results showed that compared with the blank control (Con) group, the relative expression levels of circ-ZNF652 in SMMC-7721 cells and their derived exosomes were significantly upregulated in the TGF-β1 group (*P* <0.01). Moreover, the expression of circ-ZNF652 in cells and exosomes was further increased in the TGF-β1+Exos-circ-ZNF652 group compared with the TGF-β1 group (*P* < 0.05). In contrast, there was no significant difference in circ-ZNF652 expression between the TGF-β1 + NC RNA group and the TGF-β1 group (*P* > 0.05) (Fig. 1E). These results demonstrate that TGF-β1 stimulation significantly upregulated circ-ZNF652 expression in both SMMC-7721 cells and their secreted exosomes, and that intervention with Exos-circ-ZNF652 further increased intracellular circ-ZNF652 levels in recipient HCC cells.

### Effects of TGF-β1 and Exos-circ-ZNF652 on EMT-Related Proteins

Having established that TGF-β1 upregulates circ-ZNF652 in secreted exosomes (Fig. 1), we next investigated whether these exosomes induce phenotypic changes associated with EMT in recipient HCC cells. Western blot results showed that compared with the Con group, the relative expression levels of Twist1, N-cadherin, vimentin, andMMP-9 in SMMC-7721 cells were significantly upregulated in the TGF-β1 group (Fig. 2A). Furthermore, the expression levels of these EMT-related proteins were further significantly elevated in the TGF-β1 +Exos-circ-ZNF652 group compared with the TGF-β1 group (*P* < 0.01). No significant difference in the expression of the above EMT-related proteins was observed between the TGF-β1 + NC RNA group and the TGF-β1 group (*P* > 0.05). Notably, EMT is characterized by reciprocal downregulation of epithelial markers (*e.g.*, E-cadherin) alongside mesenchymal marker elevation; thus, the observed upregulation of Twist1, N-cadherin, vimentin, and MMP-9 here is consistent with established EMT induction by TGF-β1. These results indicate that exosome-derived circ-ZNF652 from SMMC-7721 cells potentiates TGF-β1-induced mesenchymal marker expression in HCC cells. While these changes are consistent with partial EMT features, definitive confirmation requires concomitant assessment of epithelial marker loss (Fig. 2B–2E).

### Exos circ-ZNF652 Enhances TGF-β1-Induced Proliferation and Migration of SMMC-7721 Cells

Since EMT is often accompanied by metabolic shifts toward glycolysis, we next assessed whether Exos-circ-ZNF652 alter proliferative and migratory activity in SMMC-7721 cells. MTT assay results showed that compared with the Con group, the cell viability of SMMC-7721 cells was significantly increased in the TGF-β1 +Exos-circ-ZNF652 group (*P* <0.01 vs both Con group and TGF-β1 +NC RNA groups) (Fig. 3A), indicating that TGF-β1 combined with Exos circ-ZNF652 can effectively promote theproliferative activity of SMMC-7721 cells. No significant differencein cell viability was found between the TGF-β1 + NC RNA group andthe TGF-β1 group (Fig. 3A).

Consistent results were obtained from the wound healing assay (×100 magnification). At 24h after scratching, the relative migration distance of SMMC-7721 cells in the TGF-β1 +Exos-circ-ZNF652 group was significantly higher than that in all other groups (***P <0.001 vs Con group; ##P<0.01 vs TGF-β1 group and TGF-β1 +NC RNA) (Fig. 3B and 3C). The TGF-β1 group also showed a significantly higher relative migration distance compared with the Con group (**P <0.01), while no significant difference was observed between the TGF-β1 + NC RNA group and the TGF-β1 group (*P* >0.05) (Fig. 3B and 3C). These results confirm that TGF-β1 can induce the proliferation and migration of SMMC-7721 cells, and Exos circ-ZNF652 acts as a critical “amplifier” to synergistically enhance these malignant biological behaviors of HCC cells.

### Exos circ-ZNF652 Promotes Cell Cycle Progression and Inhibits Apoptosis in TGF-β1-Stimulated SMMC-7721 Cells and HepG2 Cells

Flow cytometry analysis revealed that compared with the Con group, the TGF-β1 group had a significantly higher S-phase cell proportion (28.64 ± 2.15% vs 19.32 ± 1.56%, *P* < 0.01) and a markedly lower apoptosis rate (5.21 ± 0.63% vs 12.87 ± 1.12%, *P* < 0.001). The TGF-β1 + Exos-circ-ZNF652 group showed a further significant increase in S-phase cell proportion (36.89 ± 2.58%, *P* < 0.001 vs Con group; # *P* < 0.05 vs TGF-β1 group and TGF-β1 + NC RNA) and a further remarkable decrease in apoptosis rate (2.35 ± 0.31%, *P* < 0.001 vs Con group; # *P* < 0.05 vs TGF-β1 group and TGF-β1 + NC RNA). No significant differences in S-phase cell proportion and apoptosis rate were observed between the TGF-β1 + NC RNA group and the TGF-β1 group (*P* > 0.05).

BrdU ELISA assay results showed that TGF-β1 stimulation significantly increased the DNA synthesis level of SMMC-7721 cell compared with the Con group (*P* < 0.001), and Exos-circ-ZNF652 intervention further enhanced this effect (*P* < 0.01 vs TGF-β1 group and TGF-β1 + NC RNA group)(Fig. 4A). No significant difference in DNA synthesis was found between the TGF-β1 +NC RNA group and the TGF-β1 group (Fig. 4A). Cell growth curve analysis (0−72 h) showed that the cell number of the TGF-β1+ Exos-circ-ZNF652 group was significantly higher than that of the Con group at 48 h and 72 h (*P* < 0.001), and was also significantly higher than that of the TGF-β1 group at 72 h (*P* < 0.01) (Fig. 4B). The TGF-β1 group also had a significantly higher cell number compared with the Con group at 72h (*P* < 0.01) (Fig. 4B), while no significant difference was observed between the TGF-β1 + NC RNA group and the TGF-β1 group at all time points (*P* > 0.05) (Fig. 4C).

Population doubling time (PDT) calculation showed that the PDT of the TGF-β1 +Exos-circ-ZNF652 group was the shortest (18.5 ± 1.2 h), which was significantly shorter than that of the Con group (33.3±2.1 h, *P* < 0.01) and the TGF-β1 group (25.6 ± 1.8 h, *P* < 0.01) (Fig. 4E). The PDT of the TGF-β1+ NC RNA group was not significantly different from that of the TGF-β1 group (24.9 ± 1.5 h, *P* > 0.05) (Fig. 4C). Caspase-3 activity assay showed that TGF-β1 treatment significantly inhibited Caspase-3 activity in SMMC-7721 cells (62.3±5.8% of the Con group, *P* < 0.001), and exosomalcirc-ZNF652 intervention further reduced Caspase-3 activity (41.5 ± 3.2% of the Con group, *P* <0.001 vs Con group; *P* <0.01 vs TGF-β1 group and TGF-β1 + NC RNA group) (Fig. 4G). No significant difference in Caspase-3activity was found between the TGF-β1 + NC RNA group and the TGF-β1 group (60.8 ± 5.2% of the Con group, *P* >0.05) (Fig. 4G). These results suggest that Exos-circ-ZNF652 can synergize with TGF-β1 to promote G1/S phase transition of the cell cycle and inhibit HCC cell apoptosis by reducing Caspase-3 activity, thereby significantly enhancing the proliferative capacity of SMMC-7721 cells.

In addition to SMMC-7721 cells, we investigated the effects of Exos-circ-ZNF652 in HepG2 cells. Consistent with the observations in SMMC-7721 cells, Exos-circ-ZNF652 significantly enhanced BrdU incorporation (Fig. 4B) and cell proliferation (Fig. 4D) in HepG2 cells stimulated with TGF-β1. Furthermore, the population doubling time was significantly shortened (Fig. 4F), and Caspase-3 activity was markedly reduced (Fig. 4H) in the Exos-circ-ZNF652 treated group compared to the TGF-β1 alone group. These data confirm that the pro-tumorigenic effects of Exos-circ-ZNF652 are not limited to a single cell line but are reproducible across different HCC subtypes.

### Exos circ-ZNF652 Synergizes with TGF-β1 to Enhance Glycolytic Metabolism in SMMC-7721 Cells

The TGF-β1+Exos-circ-ZNF652 co-treatment group displayed the most pronounced glycolytic phenotype. Both glucose uptake and lactate secretion in this group were significantly higher than in all other experimental groups (**P < 0.01 vs Con group and TGF-β1+NC RNA group), indicating the most active “substrate uptake-metabolite output” cycle of glycolysis (Fig. 5A and 5B). This active cycle not only provides the energy basis for rapid tumor cell proliferation but also creates an acidic tumor microenvironment conducive to tumor invasion, metastasis, and immune evasion.

The activities of both HK and LDHA peaked in the TGF-β1+Exos-circ-ZNF652 co-treatment group and were significantly higher than in the other three groups (***P < 0.001 vs Con group, ###*P* < 0.001 vs TGF-β1 group) (Fig. 5C and 5D). This indicates that Exos circ-ZNF652 can synergize with TGF-β1 to enhance the catalytic efficiency of HK and LDHA, key enzymes in the glycolytic pathway, providing a direct enzymological explanation for the observed peaks in glucose uptake and lactate secretion in the co-treatment group.

Western blot results further revealed that the TGF-β1 + Exos-circ-ZNF652 group significantly upregulated the expression levels of glucose transporter 1 (Glut-1) and hypoxia-inducible factor-1α (HIF-1α), with their expression being significantly higher than in other experimental groups (***P < 0.001 vs Con group, ### *P* < 0.001 vs TGF-β1 group) (Fig. 5E and 5F). The upregulation of Glut-1, the primary glucose transporter on the cell membrane, directly explains the significantly increased glucose uptake in the co-treatment group. The synergistic enhancement of HIF-1α expression, a core transcriptional regulator, indicates at the molecular mechanism level that Exos circ-ZNF652 can amplify the activating effect of TGF-β1 signaling on the transcriptional network of glycolysis-related genes (Fig. 5G). These protein-level changes corroborate the findings on glucose uptake, lactate secretion, and key enzyme activities, together forming a complete chain of evidence for the synergistic regulation of the glycolytic pathway in HCC cells by TGF-β1 and Exos-circ-ZNF652.

### Exos circ-ZNF652 Promotes HCC Tumor Growth *In Vivo*

In the nude mouse subcutaneous xenograft model, the tumor volume of the OE-circ group increased rapidly with time, and the tumor volume and weight of the OE-circ group were significantly higher than those of the Control group at 4 weeks (tumor volume: 1286.5 ± 105.3 mm^3^ vs 452.8 ± 42.6 mm^3^, *** *P* <0.001; tumor weight: 1.32 ± 0.11 g vs 0.48 ± 0.05 g, *** *P* < 0.001). In contrast, the KD-circ group showed significant inhibition of tumor growth compared with the Control group (tumor volume: 186.4 ± 16.5 mm^3^, *** *P* <0.001; tumor weight: 0.21 ± 0.02 g, *** *P* <0.001) (Fig. 6A and 6B). Western blot analysis of tumor tissues showed that the expression of proliferation marker Ki-67, EMT marker N-cadherin and glycolysis marker HIF-1α in the OE-circ group was significantly upregulated compared with the Control group (***P < 0.001), while their expression in the KD-circ group was significantly downregulated (**P < 0.01, *** *P* <0.001) (Fig. 6C-6F). In addition, the expression of p-AKT (downstream signaling molecules) in the OE-circ group was significantly upregulated compared with the Control group (**P < 0.01) (Fig. 6G). Biochemical detection of tumor tissue homogenates showed that the lactate content and HK activity in the OE-circ group were significantly higher than those in the Control group (***P < 0.001), and significantly lower in the KD-circ group (**P < 0.01) (Fig. 6H and 6I). These *in vivo* results further confirm that circ-ZNF652 promotes HCC tumor growth by inducing EMT and enhancing glycolysis, which is consistent with the *in vitro* experimental results.

### AKT/HIF-1α Signaling Is Required for circ-ZNF652-Mediated EMT and Glycolysis in HCC Cells

Based on the established role of AKT/HIF-1α in EMT and glycolysis and our observation of increased p-AKT/HIF-1α by TGF-β1 + Exos-circ-ZNF652 (Fig. 5E–5G), we next assessed whether this pathway mediates circ-ZNF652’s effects via rescue experiments in circ-ZNF652-overexpressing SMMC-7721 cells with AKT inhibitor (MK-2206) or HIF-1α inhibitor (KC7F2) treatment. Western blot results showed that circ-ZNF652 overexpression significantly upregulated the expression of EMT-related proteins (Twist1, Vimentin, and N-cadherin) and glycolysisrelated proteins (Glut-1, HIF-1α) compared with the vector group (***P < 0.001). However, treatment with MK-2206 or KC7F2 significantly reversed the upregulation of these proteins in circ-ZNF652-overexpressing cells (###*P* <0.001 vs OE-circ group) (Fig. 7A). There was no significant difference in the expression of these proteins between the MK-2206 treatment group and the KC7F2 treatment group (*P > 0.05) (Fig. 7B−7F). These results indicate that circ-ZNF652 is involved in EMT and glycolysis in HCC cells dependent on the AKT/HIF-1α signaling pathway, and inhibition of this pathway can reverse the oncogenic effect of circ-ZNF652.

## Discussion

This study is the first to report that exosome-derived circ-ZNF652 is associated with TGF-β1-induced EMT, and synergizes with TGF-β1 to promote cell proliferation, cell cycle progression, anti-apoptosis and glycolytic metabolism in human HCC SMMC-7721 cells [14, 18]. Our in vitro and *in vivo* results collectively confirm the oncogenic role of Exos circ-ZNF652 in HCC, and further reveal that the AKT/HIF-1α signaling pathway is the key downstream mechanism of circ-ZNF652 in regulating mesenchymal phenotype acquisition and glycolysis [20, 21]. These findings provide a novel molecular link between TGF-β1, exosomal circRNAs and metabolic reprogramming in HCC, and identify circ-ZNF652 as a potential diagnostic marker and therapeutic target for HCC [19, 22].

TGF-β1 is a classic inducer of EMT in various tumors including HCC, and its role in regulating EMT has been well documented [8, 23]. In this study, we found that TGF-β1 stimulation significantly upregulated circ-ZNF652 expression in SMMC-7721 cells and their derived exosomes, and Exos circ-ZNF652 was effectively internalized by HCC cells. Further experiments showed that Exos circ-ZNF652 significantly enhanced TGF-β1-induced upregulation of EMT-related proteins (Twist1, N-cadherin, vimentin, MMP-9). Twist1, a key EMT transcription factor, can directly bind to the E-cadherin promoter to inhibit its expression and promote the transcription of mesenchymal markers [24]. MMP-9, a matrix metalloproteinase, can degrade the extracellular matrix (ECM) and promote tumor cell invasion and metastasis [25]. Our results are consistent with the study by Li *et al*., which found that circ-ZNF652 promotes HCC cell metastasis by inducing mesenchymal marker expression [18]. Notably, we further confirmed that Exos circ-ZNF652 acts as an amplifier of TGF-β1-induced mesenchymal marker expression. A notable limitation of this study is the absence of experimental evaluation regarding the downregulation of epithelial markers (*e.g.*, E-cadherin or ZO-1), which is a requisite criterion for definitively characterizing a full EMT program. Consequently, while the observed phenotypic shifts align with partial EMT characteristics, we have conservatively described these findings as ‘mesenchymal marker induction’ throughout the manuscript. Future investigations are warranted to incorporate concurrent assessment of epithelial markers to fully delineate the EMT spectrum potentially mediated by circ-ZNF652.

Cell cycle progression and apoptosis inhibition are important biological processes for tumor cell proliferation [23]. Our study found that TGF-β1 combined with Exos circ-ZNF652 significantly increased the S-phase cell population and reduced the apoptosis rate of HCC cells, which was accompanied by a decrease in Caspase-3 activity. Caspase-3 is a key executor of apoptosis, and its inhibition is an important mechanism for tumor cell anti-apoptosis [26]. BrdU ELISA assay and cell growth curve analysis further confirmed that Exos circ-ZNF652 can enhance TGF-β1-induced DNA synthesis and cell proliferation, and shorten the population doubling time of HCC cells. These results indicate that Exos circ-ZNF652 not only enhances glycolysis to provide energy for cell proliferation, but also directly inhibits the apoptotic pathway to promote cell survival. The *in vivo* xenograft experiment further confirmed the oncogenic role of circ-ZNF652: circ-ZNF652 overexpression significantly promoted HCC tumor growth, and upregulated the expression of proliferation (Ki-67), EMT (N-cadherin) and glycolysis (HIF-1α) markers in tumor tissues. These *in vivo* results are consistent with our *in vitro* findings, and provide strong evidence for the role of circ-ZNF652 in HCC progression.

In addition to inducing EMT, TGF-β1 is also a key regulator of tumor metabolic reprogramming [12]. The Warburg effect is a hallmark of HCC, and enhanced glycolysis can provide energy and anabolic intermediates for tumor cell proliferation and metastasis [27]. In this study, we found that TGF-β1 stimulation significantly increased glucose uptake, lactate secretion, and the activity of HK and LDHA (key glycolytic enzymes) in HCC cells, and upregulated the expression of Glut-1 and HIF-1α (key glycolysis-related proteins). Exos circ-ZNF652 further enhanced these glycolytic phenotypes, which is consistent with the finding that circRNAs can regulate tumor glycolysis [28]. Glut-1 is the main glucose transporter on the cell membrane, and its upregulation directly increases glucose uptake by tumor cells [29]. HIF-1α is a core transcriptional regulator of glycolysis, which can activate the transcription of a variety of glycolysis-related genes including HK, LDHA and Glut-1 [30]. Our results suggest that Exos circ-ZNF652 enhances TGF-β1-induced glycolysis by upregulating HIF-1α expression, thereby providing energy for HCC cell proliferation and migration.

To clarify the underlying molecular mechanism, we performed a rescue experiment and found that the AKT/HIF-1α signaling pathway is required for circ-ZNF652-mediated EMT and glycolysis in HCC cells. AKT is a key serine/threonine kinase in the PI3K/AKT signaling pathway, which is frequently activated in HCC and regulates cell proliferation, EMT and glycolysis [31]. HIF-1α is a downstream target of AKT, and AKT can phosphorylate HIF-1α to enhance its stability and transcriptional activity [32]. Our results showed that inhibition of AKT or HIF-1α can reverse the upregulation of EMT and glycolysis-related proteins induced by circ-ZNF652 overexpression, indicating that circ-ZNF652 regulates EMT and glycolysis in HCC cells through the AKT/HIF-1α signaling pathway. This finding is consistent with the study by Huang *et al*., which found that TGF-β1 induces glycolysis through the Smad3/HIF-1α axis in non-small cell lung cancer [33]. Collectively, based on our *in vitro* and *in vivo* findings, we propose a novel regulatory model: TGF-β1 stimulation upregulates circ-ZNF652 expression in HCC cells, which is subsequently packaged into exosomes and secreted into the tumor microenvironment. Exosomal circ-ZNF652 is then internalized by recipient HCC cells, where it activates the AKT/HIF-1α signaling axis, thereby amplifying TGF-β1-induced EMT and glycolytic reprogramming, and ultimately driving HCC cell proliferation, migration, and *in vivo* tumor growth.

Several limitations of this study should be acknowledged and addressed in future investigations. First, we only used a single HCC cell line (SMMC-7721) *in vitro* experiments; future studies should validate our findings in other HCC cell lines (e.g., HepG2, Huh7) to improve the generalizability of the results. Second, although we performed a nude mouse xenograft experiment to verify the role of circ-ZNF652 in tumor growth, we did not investigate the role of Exos circ-ZNF652 in HCC metastasis *in vivo* (e.g., lung metastasis model); future studies should construct animal models of distant metastasis to clarify the role of Exos circ-ZNF652 in HCC metastasis. Third, we used differential centrifugation combined with a commercial kit to extract exosomes, which may contain a small amount of contaminating proteins; future studies can use density gradient centrifugation or size exclusion chromatography to obtain higher purity exosomes. Fourth, a key limitation is that we did not assess the loss of epithelial markers (e.g., E-cadherin, ZO-1), which is essential for definitively concluding a full EMT process. Our conclusions are therefore restricted to the induction of mesenchymal-associated proteins. Future studies should validate these findings by concurrently measuring both epithelial and mesenchymal markers. Fifth, we only clarified the role of the AKT/HIF-1α signaling pathway; future studies should explore the interaction network between circ-ZNF652 and miRNAs/mRNAs to further elucidate the detailed molecular mechanism of circ-ZNF652 in HCC.

Despite these limitations, our study still provides important insights into the molecular mechanism of HCC progression. We identified Exos circ-ZNF652 as a novel mediator of TGF-β1-induced EMT and glycolysis in HCC, and revealed the AKT/HIF-1α signaling pathway as its key downstream mechanism. These findings suggest that Exos circ-ZNF652 may serve as a potential non-invasive diagnostic marker for HCC (due to its stable expression in exosomes) and a novel therapeutic target (inhibition of circ-ZNF652 or its downstream AKT/HIF-1α signaling pathway may inhibit HCC progression). Future clinical translational research should focus on developing circ-ZNF652-based diagnostic probes and therapeutic drugs, which may provide new strategies for the clinical diagnosis and treatment of HCC.

## Figures and Tables

**Fig. 1:**
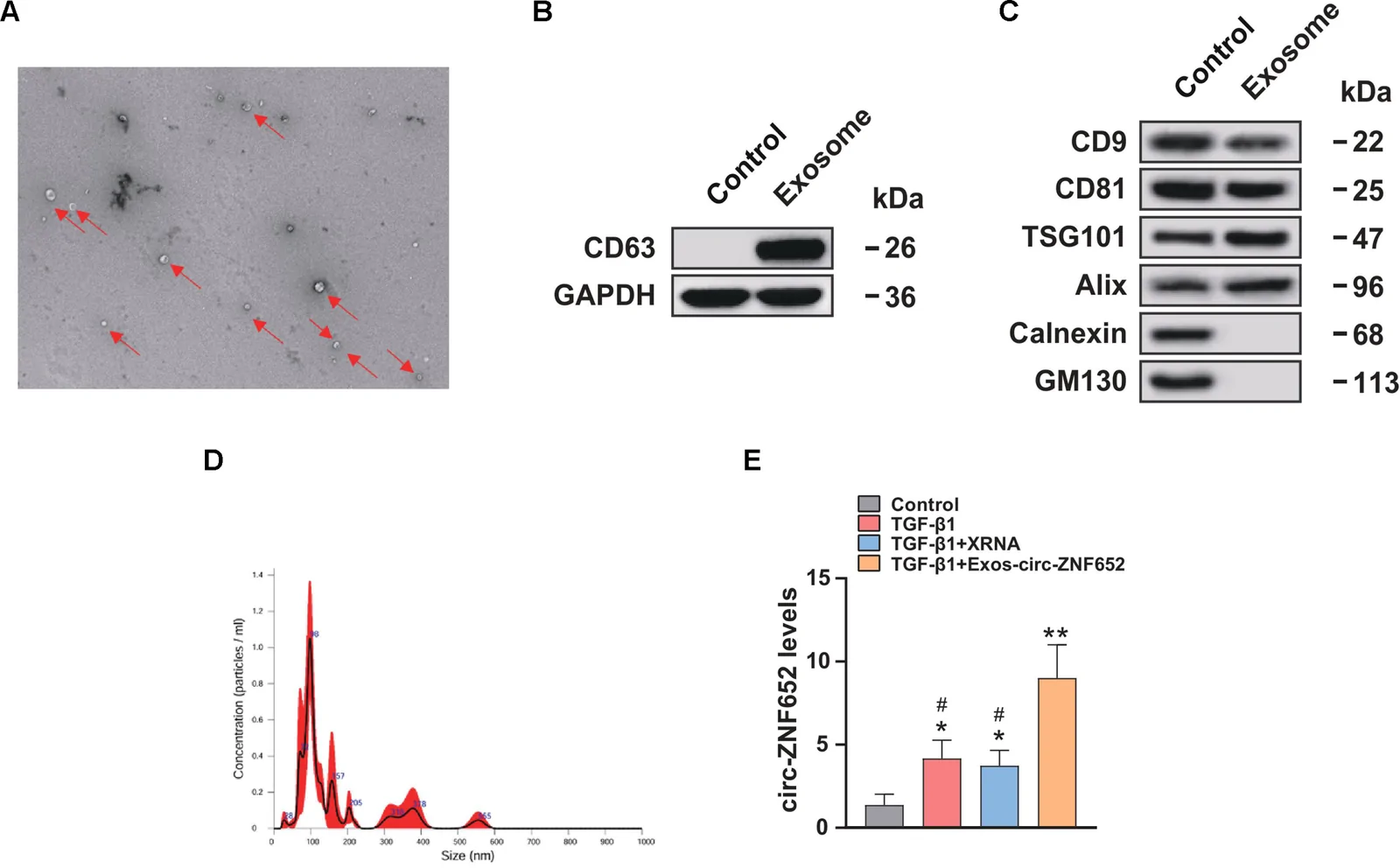
Identification and cellular uptake of exosomes from SMMC-7721 cells and circ-ZNF652 expression after TGF-β1 stimulation. (**A**)TEM images of exosomes showing typical cup-shaped structures; (**B**) Western blot analysis of exosome marker CD63 in exosome fraction and cell lysate (control); (**C**) Western blot analysis of exosomal markers (CD9, CD81, TSG101, Alix) and the negative marker Calnexin in exosome isolates and whole-cell lysates (control); (**D**) NTA analysis of exosome particle size distribution (30−150 nm); (**E**) Relative expression of circ-ZNF652 in cells and exosomes of each group detected by RT-qPCR. Data are presented as mean ± SD (n = 3 independent biological replicates). Statistical significance was assessed by one-way ANOVA with Tukey’s post-hoc test. *P < 0.05, ** *P* < 0.01 vs Con group; # *P* < 0.05 vs TGF-β1 + circ-ZNF652 group (one-way ANOVA).

**Fig. 2:**
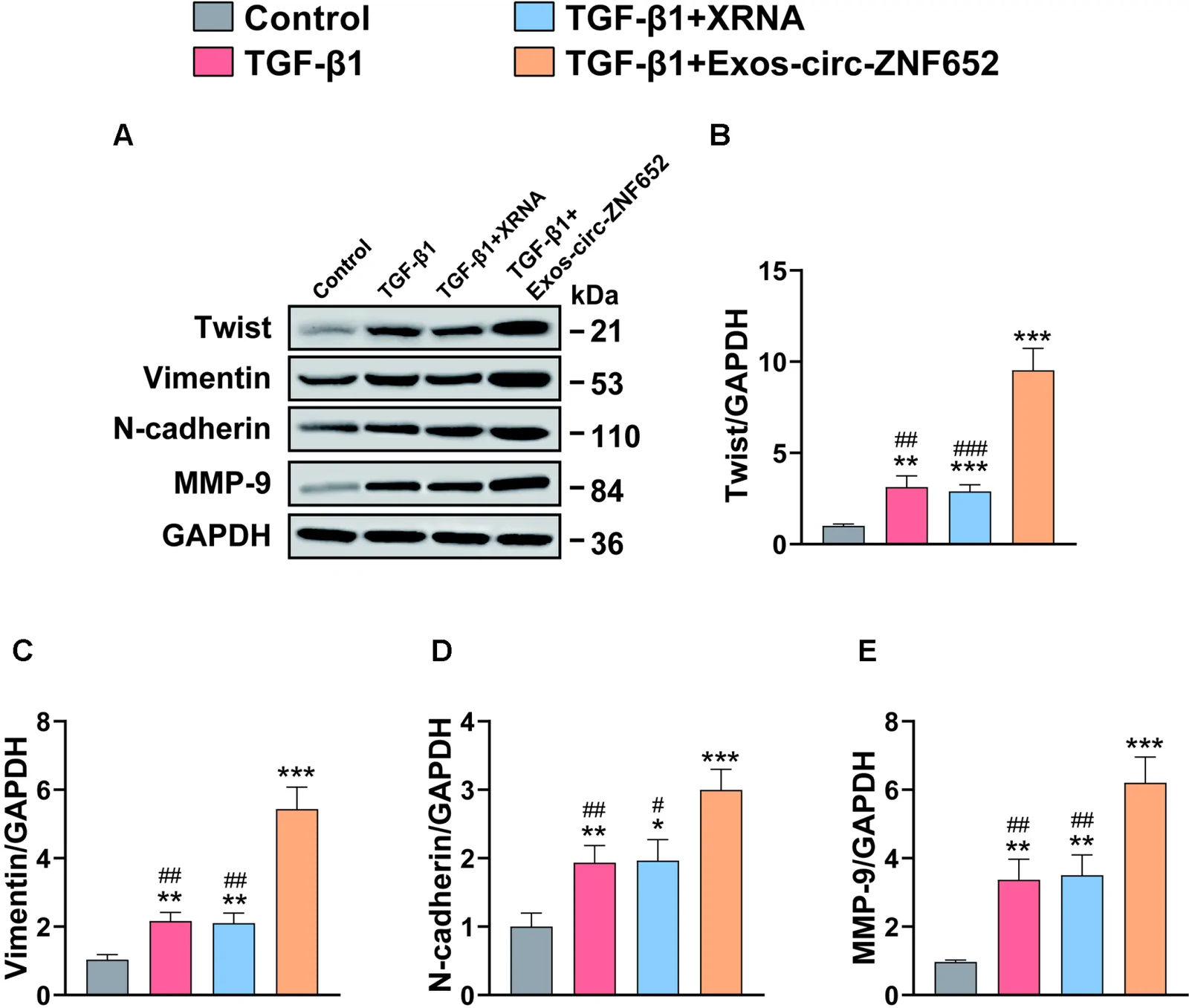
Exosomal circ-ZNF652 potentiates TGF-β1-induced upregulation of mesenchymal markers in SMMC-7721 cells. (**A**) Western blot analysis of Twist1, N-cadherin, vimentin, and MMP-9 expression in each group; Quantitative analysis of the relative gray values of mesenchymal markers (normalized to GAPDH): (**B**) Twist1, (**C**) Vimentin, (**D**) N-cadherin, (**E**) MMP-9. Data are presented as mean ± SD (n = 3 independent biological replicates). Statistical significance was assessed by one-way ANOVA with Tukey’s post-hoc test. *** *P* < 0.001 vs. the Con group; ## *P* < 0.01, ### *P* < 0.001 vs. the TGF-β1 alone group.

**Fig. 3:**
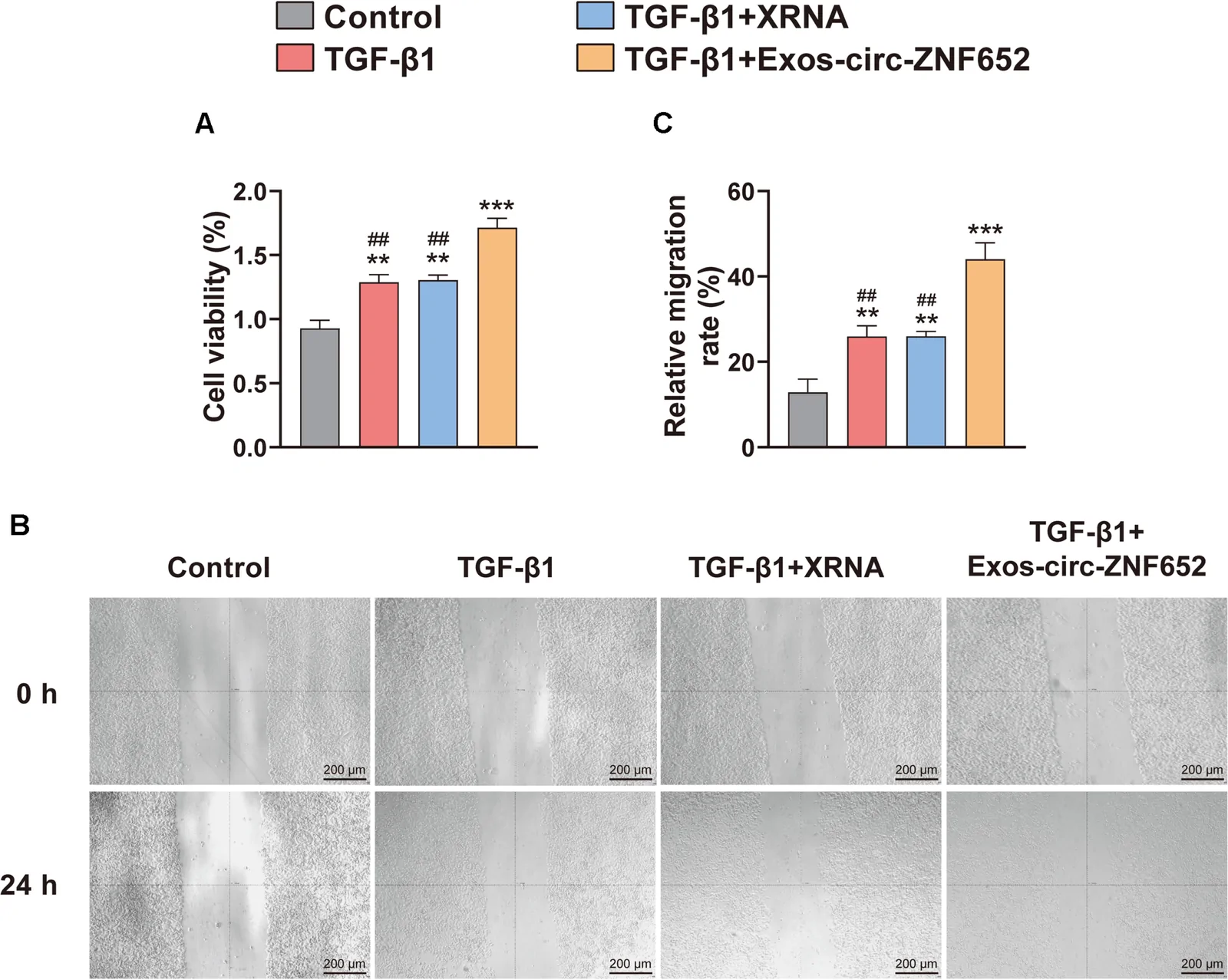
Exos circ-ZNF652 promotes TGF-β1-induced proliferation and migration of SMMC-7721 cells. (**A**) Cell viability detected by MTT assay; (**B**) Relative migration distance detected by wound healing assay (×100 magnification); (**C**) Quantitative analysis of the relative migration distance at 24 h post-scratching (scale bar = 200 μm). Data are presented as mean ± SD (n = 3 independent biological replicates). Statistical significance was assessed by one-way ANOVA with Tukey’s post-hoc test. ** *P* < 0.01, *** *P* < 0.001 vs. the Con group; ## *P* < 0.01 vs. the TGF-β1 alone group.

**Fig. 4:**
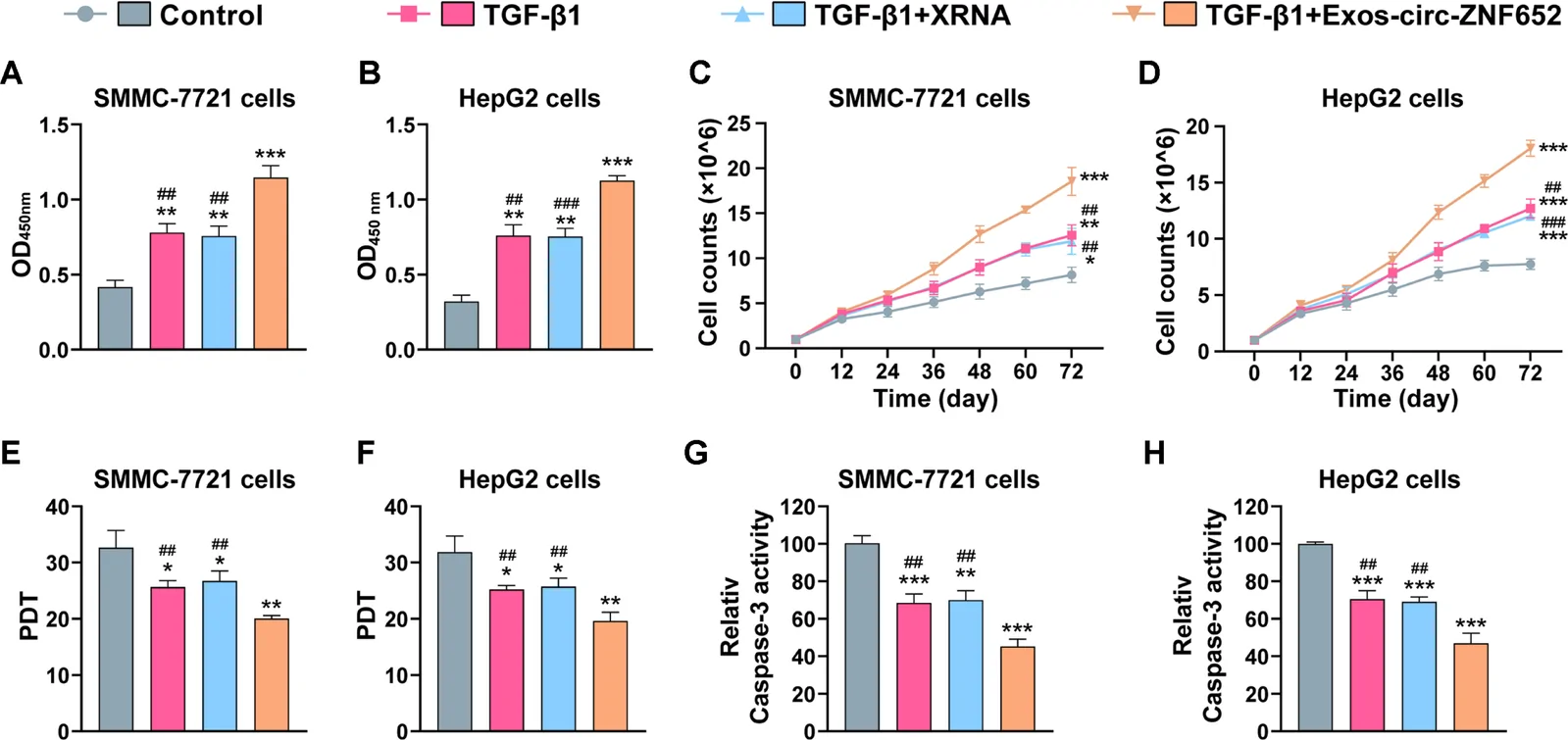
Exos circ-ZNF652 accelerates cell cycle progression and inhibits apoptosis in TGF-β1-stimulated SMMC-7721 cells and HepG2 cells. (**A-B**). BrdU ELISA assay for cell proliferation activity (DNA synthesis) in SMMC-7721 and HepG2 cells; (**C-D**) Cell growth curve (0−72 h) of SMMC-7721 and HepG2 cells detected by trypan blue staining; (**E-F**) Population doubling time (PDT) calculation of each group in SMMC-7721 and HepG2 cells; (**G-H**) Caspase-3 activity of SMMC-7721 and HepG2 cells detected by colorimetric assay (relative to Con group). Data are presented as mean ± SD (n = 3 independent biological replicates). Statistical significance was assessed by one-way ANOVA with Tukey’s post-hoc test. * *P* < 0.05, ** *P* < 0.01, *** *P* < 0.001 vs Con group; ## *P* < 0.01 vs TGF-β1 + Exos-circ-ZNF652 group (one-way ANOVA).

**Fig. 5:**
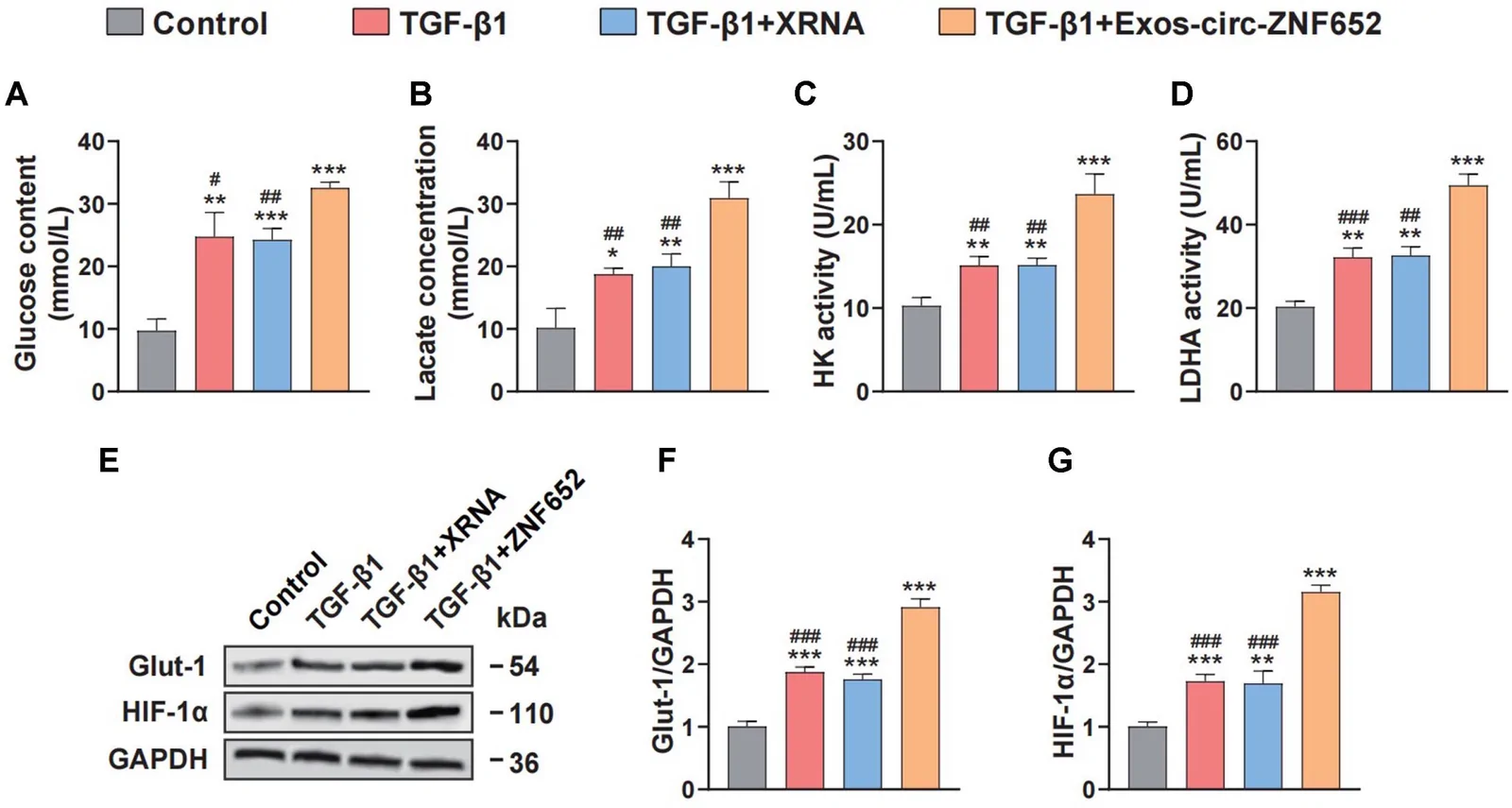
Exos circ-ZNF652 synergizes with TGF-β1 to enhance glycolytic metabolism in SMMC-7721 cells. (**A**) Glucose uptake of each group; (**B**) Lactate secretion of each group; (**C**) HK activity of each group; (**D**) LDHA activity of each group; (**E**) Western blot analysis of Glut-1 and HIF-1α expression; Quantitative analysis of (**F**) Glut-1 and (**G**) HIF-1α relative expression (normalized to GAPDH). Data are presented as mean ± SD (n = 3 independent biological replicates). Statistical significance was assessed by one-way ANOVA with Tukey’s post-hoc test. *** *P* < 0.001 vs Con group; ### *P* < 0.001 vs TGF-β1 group (one-way ANOVA).

**Fig. 6:**
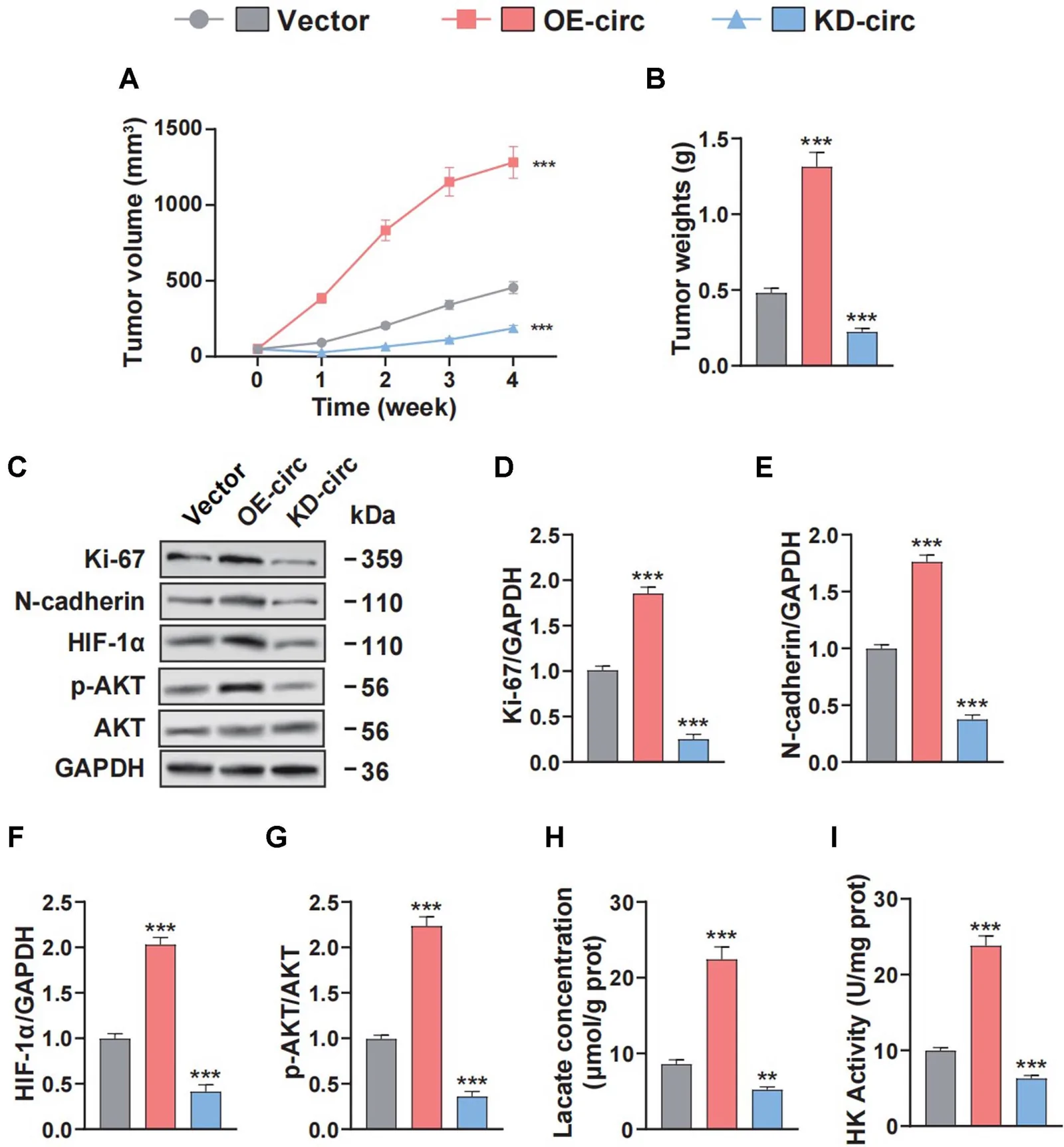
Circ-ZNF652 promotes HCC tumor growth in a nude mouse subcutaneous xenograft model. (**A**) Tumor growth curve of each group (tumor volume measured weekly for 4 weeks); (**B**) Tumor weight of each group at 4 weeks post-inoculation; (**C**) Western blot analysis of Ki-67, N-cadherin, HIF-1α, total AKT, and p-AKT in tumor tissues; Quantitative analysis of the relative protein expression (normalized to GAPDH or total AKT): (**D**) Ki-67, (**E**) N-cadherin, (**F**) HIF-1α, (**G**) p-AKT/AKT ratio; (**H**) Lactate content in tumor tissues; (**I**) HK activity in tumor tissues. Data are presented as mean ± SD (n = 6 mice per group). Statistical significance was assessed by one-way ANOVA with Tukey’s post-hoc test. ** *P* < 0.01, ***P < 0.001 vs. the Control group.

**Fig. 7:**
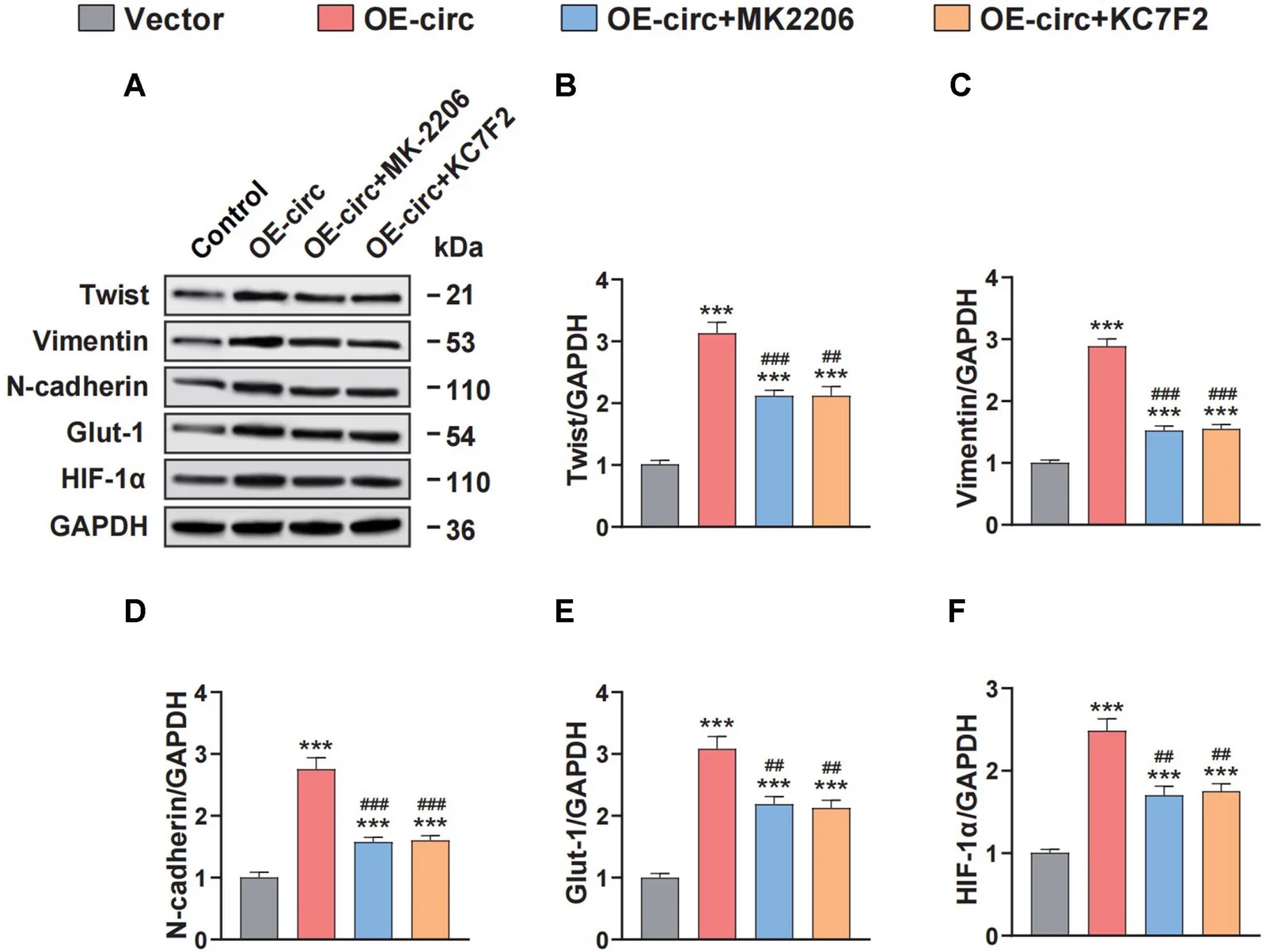
The AKT/HIF-1α signaling axis is required for circ-ZNF652-induced EMT and glycolytic reprogramming in HCC cells (rescue assay). (**A**) Western blot analysis of EMT-related proteins (Twist1, N-cadherin, vimentin) in circ-ZNF652-overexpressing (OE-circ) cells treated with MK-2206 or KC7F2; (**B**) Western blot analysis of glycolysis-related proteins (Glut-1, HIF-1α) in the above groups; (C-E) Quantitative analysis of the relative expression of EMT-related proteins: (**C**) Twist1, (**D**) N-cadherin, (**E**) Vimentin; (**F-G**) Quantitative analysis of the relative expression of glycolysis-related proteins: (**F**) Glut-1, (**G**) HIF-1α. Data are presented as mean ± SD (n = 3 independent biological replicates). Statistical significance was assessed by one-way ANOVA with Tukey’s post-hoc test. *** *P* < 0.001 vs. the Vector group; ### *P* < 0.001 vs. the OE-circ group.

## References

[1] Hwang SY, Danpanichkul P, Agopian V, Mehta N, Parikh ND, Abou-Alfa GK (2025). Hepatocellular carcinoma: updates on epidemiology, surveillance, diagnosis and treatment. Clin. Mol. Hepatol..

[2] Harimoto N, Tsukagoshi M, Seki T, Hoshino K, Hagiwara K, Ishii N (2024). Predictors for early recurrence beyond up-to-7 or distant metastasis after hepatocellular carcinoma resection: proposal for borderline resectable HCC. Int. J. Clin. Oncol..

[3] Gao W, Wang J, Xu Y, Yu H, Yi S, Bai C (2024). Research progress in the metabolic reprogramming of hepatocellular carcinoma (Review). Mol. Med. Rep..

[4] Solhi R, Pourhamzeh M, Zarrabi A, Hassan M, Mirzaei H, Vosough M (2024). Novel biomarkers for monitoring and management of hepatocellular carcinoma. Cancer Cell Int..

[5] Chan YT, Zhang C, Wu J, Lu P, Xu L, Yuan H (2024). Biomarkers for diagnosis and therapeutic options in hepatocellular carcinoma. Mol. Cancer.

[6] Yüregir Y, Kaçaroğlu D, Yaylacı S (2024). Regulation of Hepatocellular Carcinoma Epithelial-Mesenchymal Transition Mechanism and Targeted Therapeutic Approaches. Adv. Exp. Med. Biol..

[7] Xia H, Chen J, Shi M, Gao H, Sekar K, Seshachalam VP (2015). EDIL3 is a novel regulator of epithelial-mesenchymal transition controlling early recurrence of hepatocellular carcinoma. J. Hepatol..

[8] Zhuang X, Wang C, Ge Z, Wu M, Chen M, Chen Z (2025). MICAL1 Mediates TGF-β1-Induced Epithelial-to-Mesenchymal Transition and Metastasis of Hepatocellular Carcinoma by Activating Smad2/3. Cell Biochem. Biophys..

[9] Huang W, Yu D, Wang M, Han Y, Lin J, Wei D (2020). ITGBL1 promotes cell migration and invasion through stimulating the TGF-β signalling pathway in hepatocellular carcinoma. Cell Prolif..

[10] Zhang C, Zhou W, Xu H, Xu J, Li J, Liu X (2025). Cancer-associated fibroblasts promote EGFR-TKI resistance via the CTHRC1/glycolysis/H3K18la positive feedback loop. Oncogene.

[11] Liu F, Wang Q, Wang Z, Zhang S, Ni Q, Chang H (2024). ETV4 promotes the progression of cholangiocarcinoma by regulating glycolysis via the TGF-β signaling. Transl.Oncol..

[12] Yang Y, Ren P, Liu X, Sun X, Zhang C, Du X (2022). PPP1R26 drives hepatocellular carcinoma progression by controlling glycolysis and epithelial-mesenchymal transition. J. Exp. Clin. Cancer Res..

[13] Xu HZ, Lin XY, Xu YX, Xue HB, Lin S, Xu TW (2023). An emerging research: the role of hepatocellular carcinoma-derived exosomal circRNAs in the immune microenvironment. Front. Immunol..

[14] Liu Z, Wang Y, Wang Y, Zhang Y (2026). Exosomal circRNAs in hepatocellular carcinoma: Implications for the development and therapeutic resistance of hepatocellular carcinoma (Review). Int. J. Oncol..

[15] Zhao Y, Yao J (2022). The Biological Functions and Clinical Values of Exosomal Circular RNAs in Hepatocellular Carcinoma. Front. Oncol..

[16] Tonon F, Grassi C, Tierno D, Biasin A, Grassi M, Grassi G (2024). Non-Coding RNAs as Potential Diagnostic/Prognostic Markers for Hepatocellular Carcinoma. Int. J.Mol. Sci..

[17] Wang L, Tong X, Zhou Z, Wang S, Lei Z, Zhang T (2018). Circular RNA hsa_circ_0008305 (circPTK2) inhibits TGF-β-induced epithelial-mesenchymal transition and metastasis by controlling TIF1γ in non-small cell lung cancer. Mol. Cancer.

[18] Li Y, Zang H, Zhang X, Huang G (2020). Exosomal Circ-ZNF652 Promotes Cell Proliferation, Migration, Invasion and Glycolysis in Hepatocellular Carcinoma via miR-29a-3p/GUCD1 Axis. Cancer Manag. Res..

[19] Zhang H, Pei S, Li J, Zhu J, Li H, Wu G (2024). Insights about exosomal circular RNAs as novel biomarkers and therapeutic targets for hepatocellular carcinoma. Front.Pharmacol..

[20] Wang J, Shi J, Mi L, Zhao M, Han G, Yin F (2025). Aberrant activation of the PI3K/AKT/HIF-1α pathway promotes glycolysis and lenvatinib resistance in liver cancer. Mol.Med. Rep..

[21] Navarro C, Ortega Á, Santeliz R, Garrido B, Chacín M, Galban N (2022). Metabolic Reprogramming in Cancer Cells: Emerging Molecular Mechanisms and Novel Therapeutic Approaches. Pharmaceutics.

[22] Tian H, Zhu X, Lv Y, Jiao Y, Wang G (2020). Glucometabolic Reprogramming in the Hepatocellular Carcinoma Microenvironment: Cause and Effect. Cancer Manag. Res..

[23] Gungor MZ, Uysal M, Senturk S (2022). The Bright and the Dark Side of TGF-β Signaling in Hepatocellular Carcinoma: Mechanisms, Dysregulation, and Therapeutic Implications. Cancers (Basel).

[24] Meng J, Chen S, Han JX, Qian B, Wang XR, Zhong WL (2018). Twist1 Regulates Vimentin through Cul2 Circular RNA to Promote EMT in Hepatocellular Carcinoma. Cancer Res..

[25] Cui Q, Wang X, Zhang Y, Shen Y, Qian Y (2023). Macrophage-Derived MMP-9 and MMP-2 are Closely Related to the Rupture of the Fibrous Capsule of Hepatocellular Carcinoma Leading to Tumor Invasion. Biol. Proced Online.

[26] Han R, Chen X, Li Y, Zhang S, Li R, Lu L (2019). MicroRNA-34a suppresses aggressiveness of hepatocellular carcinoma by modulating E2F1, E2F3, and Caspase-3. Cancer Manag. Res..

[27] Zhou Y, Lin F, Wan T, Chen A, Wang H, Jiang B (2021). ZEB1 enhances Warburg effect to facilitate tumorigenesis and metastasis of HCC by transcriptionally activating PFKM. Theranostics.

[28] Zheng J, Yan X, Lu T, Song W, Li Y, Liang J (2023). CircFOXK2 promotes hepatocellular carcinoma progression and leads to a poor clinical prognosis via regulating the Warburg effect. J. Exp. Clin. Cancer Res..

[29] Dong F, He K, Zhang S, Song K, Jiang L, Hu LP (2024). SSRI antidepressant citalopram reverses the Warburg effect to inhibit hepatocellular carcinoma by directly targeting GLUT1. Cell Rep..

[30] Shan Q, Yin L, Zhan Q, Yu J, Pan S, Zhuo J (2024). The p-MYH9/USP22/HIF-1α axis promotes lenvatinib resistance and cancer stemness in hepatocellular carcinoma. Signal Transduct. Target. Ther..

[31] Li M, Chen J (2026). STOML2 promotes hepatocellular carcinoma cell proliferation, invasion and migration by activating the PI3K/AKT signaling pathway (Review). Oncol.Lett..

[32] Gómez-Virgilio L, Velazquez-Paniagua M, Cuazozon-Ferrer L, Silva-Lucero MD, Gutierrez-Malacara AI, Padilla-Mendoza JR (2024). Genetics, Pathophysiology, and Current Challenges in Von Hippel-Lindau Disease Therapeutics. Diagnostics (Basel).

[33] Huang Y, Chen Z, Lu T, Bi G, Li M, Liang J (2021). HIF-1α switches the functionality of TGF-β signaling via changing the partners of smads to drive glucose metabolic reprogramming in non-small cell lung cancer. J. Exp. Clin. Cancer Res..

